# Gold–Mercury–Platinum Alloy for Light-Enhanced Electrochemical Detection of Hydrogen Peroxide

**DOI:** 10.3390/s25010135

**Published:** 2024-12-29

**Authors:** Yunping Wei, Runze Li, Meng Lin

**Affiliations:** Center for Experimental Chemistry Education of Shandong University, School of Chemistry and Chemical Engineering, Shandong University, Jinan 250100, China; ypwei@sdu.edu.cn (Y.W.); 202332256@mail.sdu.edu.cn (R.L.)

**Keywords:** alloys, electrochemical detection, light enhancement, amalgam, galvanic replacement reaction

## Abstract

In this study, a simple and easy synthesis strategy to realize the modification of AuHgPt nanoalloy materials on the surface of ITO glass at room temperature is presented. Gold nanoparticles as templates were obtained by electrochemical deposition, mercury was introduced as an intermediate to form an amalgam, and then a galvanic replacement reaction was utilized to successfully prepare gold–mercury–platinum (AuHgPt) nanoalloys. The obtained alloys were characterized by scanning electron microscopy, UV–Vis spectroscopy, X-ray photoelectron spectroscopy and X-ray diffraction techniques. The electrochemical sensing performance of the AuHgPt-modified electrode for hydrogen peroxide was evaluated by cyclic voltammetry and chronoamperometry. Under light conditions, the AuHgPt-modified electrode exhibited a desirable current response in the detection of hydrogen peroxide due to the synergistic effect of the localized surface plasmon resonance effect inherent in gold nanoparticles, and this synergistic effect improved the sensitivity of hydrogen peroxide detection. Meanwhile, the AuHgPt-modified electrode also exhibited better stability and reproducibility, which makes the modified electrode have great potential for various applications in the field of electrochemical sensing.

## 1. Introduction

Localized surface plasmon resonance is the collective oscillation of free electrons on the surface of a metal nanostructure when irradiated with light of a specific wavelength; this enhances the local electric field and generates high-energy electrons and holes, leading to the strong absorption and scattering characteristics of metal nanostructures for that specific wavelength of light [1]. The LSPR effect often occurs in smaller nanoparticles, especially for precious metals such as gold and silver nanostructures [2]. The effect leads to further amplification of the electric field strength and enhancement of the optical signal by the generation of high-energy charge carriers, thus making nanoparticles widely used in technologies such as optoelectronic devices [3,4]. In electrochemical sensors based on LSPR enhancement, noble metal nanostructures (e.g., gold nanoparticles, gold nanorods, silver nanoparticles, etc.) are often used as the photoelectrical actuators of the sensors. When these nanostructures are exposed to light, the occurrence of the LSPR effect excites the generation of thermal charge carriers to form photocurrents, which accelerates the electrochemical reaction and improves the electrochemical performance of the sensors [5,6]. For instance, Xia and co-authors demonstrated that the LSPR properties of gold nanoparticles significantly increased the rate of electrocatalytic oxidation of glucose [7]. CTAB-coated gold nanorod-modified glassy carbon electrodes can be used for photoelectrochemical examination of hydrogen peroxide and detection of trace hydrogen peroxide levels released by cervical cancer cells [8]. The performance of this biosensor was significantly improved under LSPR excitation, which was mainly attributed to the enhancement of the electrocatalytic activity of the active material by the thermal charge carriers generated during the plasma decay process. It was also found that the electrocatalytic activity was directly related to the wavelength and intensity of light.

It is well known that multimetallic nanostructures, especially in the form of alloys, have superior electrocatalytic properties and higher electron transport rates than monometallic nanomaterials [9,10]. However, the direct formation of multimetallic nanoalloys from noble metals often requires more stringent conditions due to thermodynamic limitations. The galvanic replacement reaction can be used to synthesize multimetallic alloy materials due to mild reaction conditions [11], but this method requires a certain potential difference between the metals involved in the reaction. In the case of gold–platinum alloys, the potential difference between these two noble metals is very close to each other, making it more difficult to realize the synthetic preparation of the alloys. Therefore, the introduction of intermediates such as copper or nickel to synthesize precious metal nanoalloys with similar potential differences using the galvanic replacement reaction is a more feasible option [12,13]. However, this method still requires a high calcination temperature [14]. In our previous study, we explored the use of mercury (Hg) as an intermediate and successfully synthesized hollow gold-based noble metal nanoalloys using the galvanic replacement reaction at room temperature, which exhibited enhanced electrocatalytic performance in an electrochemically catalyzed hydrogen evolution reaction [15].

Hydrogen peroxide is an active molecule important in biological, chemical and environmental fields. In biological systems, it plays a role in processes such as cell signaling, immune response and metabolism [16]. In the chemical industry, it is a common oxidizing agent. In environmental monitoring, its concentration is a key indicator of the degree of pollution. Therefore, the accurate and sensitive detection of hydrogen peroxide is of paramount significance. Traditional hydrogen peroxide detection methods include fluorescence, chemiluminescence and electron spin resonance [17,18,19], which usually require expensive instrumentation and more cumbersome pre-treatment, and have certain limitations. In recent years electrochemical detection methods, especially enzyme-free sensors, have become common tools for hydrogen peroxide detection due to their high sensitivity, rapid response, simple operation and cost-effectiveness [20]. The integration of light enhancement technology with electrochemical detection further improves the sensing performance of electrochemical sensors and provides a new research avenue for highly sensitive and selective line detection of hydrogen peroxide.

Based on previous studies, this study aimed to prepare gold-based nanoalloys directly on the surface of indium–tin–oxide (ITO) glass under mild conditions. Herein, gold nanoparticles were first electrodeposited onto the surface of ITO glass, and after mercury was reduced to the gold nanoparticles’ surface to form a gold amalgam, platinum atoms and gold nanoparticles were combined and modified onto the surface of ITO glass through a galvanic replacement reaction to synthesize gold–mercury–platinum (AuHgPt) nanoalloys. In addition to the structural and compositional characterization of the nanoalloys, the electrochemical sensing response of the nanoalloys to hydrogen peroxide under the presence/absence of light was also investigated.

## 2. Materials and Methods

### 2.1. Chemicals

Chloroauric acid (HAuCl_4_·3H_2_O), potassium chloride (KCl), chloroplatinic acid (H_2_PtCl_6_), 30 wt% solution of hydrogen peroxide, ascorbic acid, glucose, uric acid and adenine were obtained from Sinopharm Chemical Reagent Co., Ltd. (Shanghai, China). Mercury ion (Hg (II)) standard solution (100 mg/L) was obtained from Sigma-Aldrich (USA). A phosphate buffer solution at pH 7.4 was prepared using a combination of NaH_2_PO_4_ and Na_2_HPO_4_, serving as the supporting electrolyte. The solvent used in the experiment was ultrapure water with a resistivity of 18.25 MΩ·cm.

### 2.2. Instruments

X-ray diffraction (XRD) measurements were conducted using a Rigaku Dmax 2000 X-ray diffractometer equipped with graphite monochromatic Cu-Kα radiation (Smart Lab XRD, Tokyo, Japan). The composition and chemical state of the samples were analyzed by X-ray photoelectron spectroscopy (XPS) using an Escalab Xi+ system from Thermo Fisher Scientific (Waltham, MA, USA) Co., Ltd. All electrochemical experiments were carried out with a CHI 750 Electrochemical Workstation (CH Instrument, Shanghai, China). A standard three-electrode configuration was employed throughout the measurements, comprising a platinum plate as the counter electrode, the modified ITO glass as the working electrode and a saturated calomel electrode (SCE) as the reference electrode. The light source was from a 300 W xenon lamp (Perfect Light, PLS-SXE300UV, Beijing, China).

### 2.3. Synthesis of AuHgPt/ITO Electrode

Initially, gold nanoparticles were deposited directly onto the surface of indium–tin–oxide (ITO) glass using an electrochemical deposition method. The deposition process employed cyclic voltammetry within a voltage range of 0.2 V to −1.0 V. Prior to the deposition, the ITO glass was immersed in a piranha solution for 30 min, followed by three cycles of ultrasonic cleaning with water and ethanol, respectively. The electrodeposition solution was prepared by mixing chloroauric acid and potassium chloride solutions with final concentrations of 24 mM and 0.1 M, respectively. Following the completion of the deposition process, the resulting Au/ITO was rinsed with water, dried, and set aside for further use.

Mercury was deposited onto the gold nanoparticles in an electrodeposition solution containing mercury (1.6 mg/mL) and potassium chloride (0.1 M) using chronoamperometry (−0.3 V). In this process, the electrodeposited mercury combined with gold to form an amalgam, obtaining an AuHg-modified electrode. Subsequently, the AuHg-modified ITO electrodes were immersed in a chloroplatinic acid solution (19 mM) for 12 h. The following reactions occurred during this experiment process [15]: 3 Hg + [PtCl_6_]^2−^ = Pt + Hg^2+^ + Hg_2_Cl_2_ + 4 Cl^−^. After immersion, the nanomaterials were rinsed thoroughly to obtain the AuHgPt nanoalloy-modified ITO electrode.

### 2.4. Electrochemical Measurements

The electrochemical methods used in the fabrication of nanoalloys and evaluation of photoelectrochemical properties include cyclic voltammetry and chronoamperometry. The current response of nanoalloy-modified electrodes under light conditions was investigated by applying different voltages. In the study of the catalytic reaction of hydrogen peroxide at the electrode, the classical three-electrode system was used and a phosphate buffer solution with pH 7.4 was designated as the electrolyte. Hydrogen peroxide was gradually added to the electrolyte to obtain the trend of the response current of the modified electrode. The influence of the modified electrode on the electrochemical response towards hydrogen peroxide under the light condition was investigated in combination with the LSPR effect of the nanoalloys.

In addition, the anti-interference capabilities of the electrode were assessed. Specifically, we investigated the electrochemical response of the modified electrode to hydrogen peroxide in the presence of uric acid, ascorbic acid, glucose and adenine, thereby comprehensively evaluating the sensing performance of the electrode in an environment of potentially interfering substances.

## 3. Results and Discussion

### 3.1. Structural and Morphological Characterization

**Figure 1:**
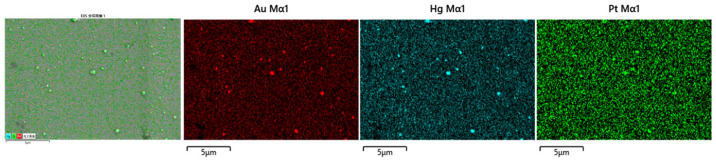
EDS elemental mapping results for AuHgPt nanoalloy.

The AuHgPt nanoalloys were obtained by a galvanic replacement reaction between platinum ions and the AuHg alloys obtained through direct electrodeposition onto ITO glass. To ascertain the structural properties of the nanoalloys, energy dispersive X-ray (EDX) elemental analysis was used to determine the elemental composition and distribution of the nanoalloys. As depicted in Figure 1, gold, mercury and platinum elements were uniformly distributed on the modified electrodes, thus confirming the successful formation of the alloy. It can also be seen from the figure that some larger particles were generated, which may be caused by the fact that the surface of the ITO electrode was not thoroughly cleaned. However, the appearance of larger particles did not affect the subsequent characterization and performance studies.

**Figure 2:**
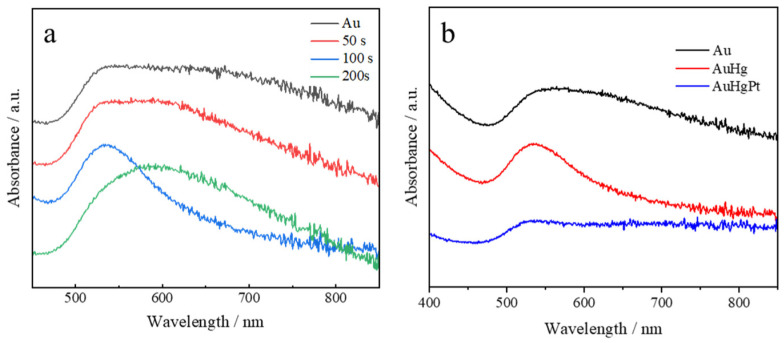
UV–Vis spectra of (**a**) gold-based nanoalloys with different Hg electrochemical deposition times and (**b**) UV–Vis absorption spectra of electrochemically deposited gold nanoparticles, AuHg and AuHgPt nanoalloys.

Localized surface plasmon resonance serves as a straightforward and effective technique for characterizing metal nanoparticles within a dielectric matrix, wherein the collective oscillations of both embedded and host nanoparticles are influenced. During the experiments, the effect of mercury deposition times of 50 s, 100 s and 200 s on the UV–Vis absorption of the alloys was investigated (Figure 2a). The UV–Vis absorption peaks of the gold nanoparticles exhibited a broader profile, suggesting a lack of uniformity in particle size and indicating poor size regularity. Changes in the particle size of the nanoalloys were observed from the UV–Vis absorption after mercury deposition. This was attributed to factors such as the surface tension of liquid mercury, as when the amount of deposited mercury increased, it led to the formation of a more homogeneous spherical morphology of the nanoparticles, and the nanoalloy particles became relatively homogeneous in size. However, when more mercury was deposited, more nanoalloys would aggregate, leading to the broadening of the UV–Vis absorption peak and red-shifting of the maximum absorption peak. Therefore, based on the above experimental results, the deposition time of mercury was selected to be 100 s. Figure 2b shows that the shape of the UV–visible absorption peaks and the location of the maximum absorption peaks of the nanoalloys were essentially the same as those of gold after the substitution of mercury by platinum. However, the intensity of absorption was reduced, but the results still illustrate that the particle size and morphology of the nanoalloy did not change significantly after the replacement of mercury by platinum.

**Figure 3:**
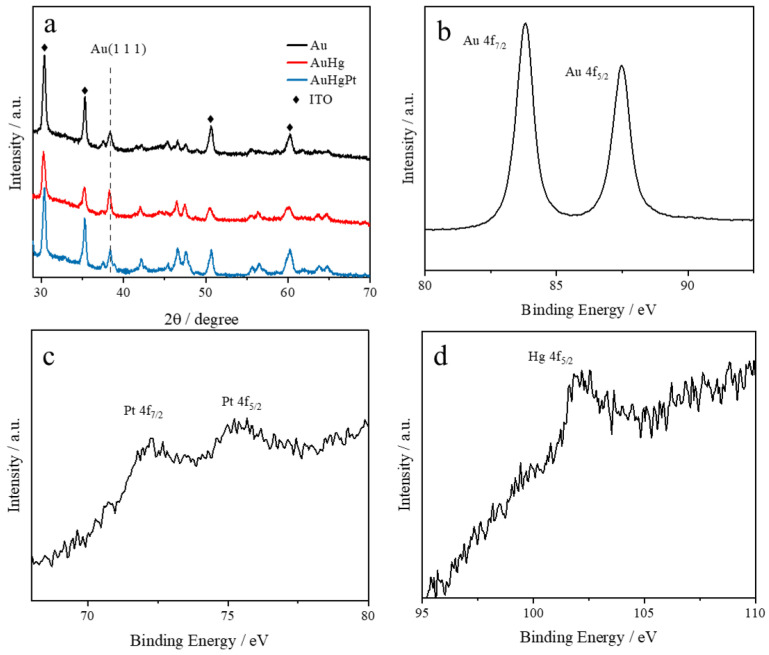
XRD patterns of the AuHgPt alloys (**a**) and high-resolution XPS spectra of Au 4f, Pt 4f and Hg 4f (**b**–**d**).

Figure 3a presents the X-ray diffraction patterns of the nanoalloys at different stages of the synthesis process. After the deposition of mercury, a notable shift in the diffraction peak position of the Au (111) crystal plane was observed, changing from 38.40° to 38.28° [21]. This shift signifies the formation of an Au amalgam. After the replacement reaction involving platinum, observations indicated that the crystal surface reverted to its original state. This phenomenon suggests that the relatively small amount of deposited and displaced platinum had little effect on the lattice structure of gold.

To further elucidate the elemental states of the AuHgPt nanoalloys, the X-ray photoelectron spectroscopy spectra for Au 4f, Pt 4f and Hg 4f are illustrated in Figure 3b–d. The gold can be observed in its metallic form, displaying two distinct peaks at 83.8 eV and 87.5 eV, which correspond to the Au^0^ 4f_7/2_ and Au^0^ 4f_5/2_ states, respectively [22]. The binding energy difference of 3.70 eV between these characteristic peaks suggests the presence of a HgPt alloy [23]. Additionally, the diffraction peak at 102.59 eV is associated with the characteristic peak of Hg^0^ 4f_5/2_ [24]. The characteristic peaks at 72.20 eV and 75.38 eV are assigned to the Pt, specifically Pt^0^ 4f_7/2_ and Pt^0^ 4f_5/2_, respectively [25]. The results show that the 4f binding energies of Au and Pt in AuHgPt alloys were shifted in the phase direction compared to pure Au and pure Pt, indicating the formation of Au–Pt alloys in the composites [23]. Moreover, the presence of mercury in the alloy indicates that a portion of the mercury was incorporated into the gold particles during the preparation of AuHg by electrodeposition and was not replaced by platinum during the galvanic replacement reaction. Thus, the results obtained from X-ray diffraction and X-ray photoelectron spectroscopy provide a comprehensive insight into the formation of AuHgPt alloys.

### 3.2. Electrochemical Characterization

**Figure 4:**
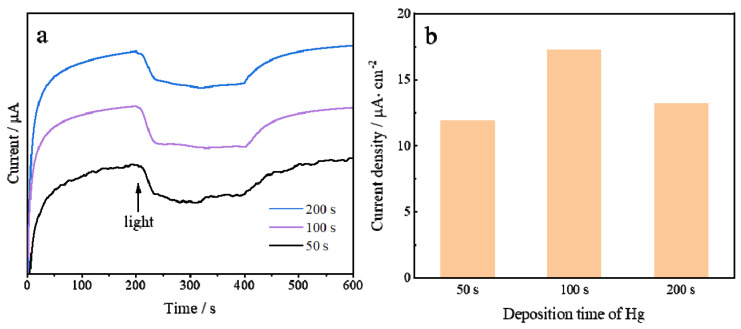
(**a**) light-enhanced current response of AuHgPt/ITO electrodes prepared with different Hg deposition times (50 s, 100 s, 200 s). (**b**) Bar graphs of current changes.

The light-enhanced electrochemical response of AuHgPt nanoalloy-modified electrodes prepared with different Hg deposition times was investigated. In the Hg deposition process, three different deposition times of 50 s, 100 s and 200 s were investigated. As can be observed in Figure 4, the current change of the AuHgPt alloy-modified electrode prepared at 100 s Hg deposition time reaches the maximum under the introduction of light, which is in high agreement with the UV absorption results obtained previously.

**Figure 5:**
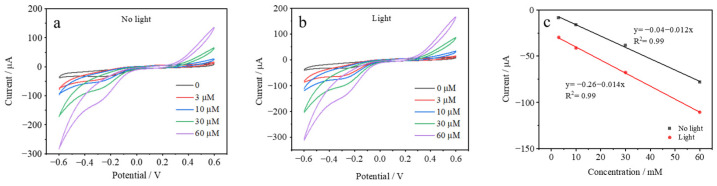
CVs of the AuHgPt/ITO in 0.1 M phosphate buffer solution containing different concentrations of hydrogen peroxide (**a**) without and (**b**) with light illumination. (**c**) Plots of peak currents vs. hydrogen peroxide concentration.

The AuHgPt/ITO electrode was further examined in depth to investigate the current response in the presence of different concentrations of hydrogen peroxide in solution and with/without the addition of light illumination. As shown in Figure 5, the cyclic voltammetric behavior of the electrode under different conditions is presented. The cyclic voltammetric curves of the electrodes with different concentrations of hydrogen peroxide increase with the increase in the hydrogen peroxide concentration in the presence/absence of light, which indicates that the AuHgPt-modified electrodes effectively enhance the electrochemical activity of hydrogen peroxide. Meanwhile, in combination with the trends of hydrogen peroxide concentration and current, the introduction of light enhances the trend of current change and the slope of the corresponding curve increases, which implies that light has a positive effect on the catalysis of hydrogen peroxide by the AuHgPt nanoalloys. The reason for this is that the alloys contain gold nanomaterials, and the light can stimulate the local surface plasmon resonance effect. The above results indicate that the optical properties of AuHgPt nanoalloys allow for light-assisted enhancement of the response current to hydrogen peroxide.

**Figure 6:**
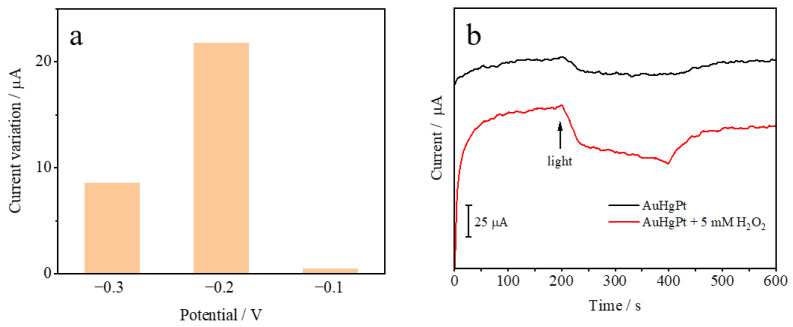
(**a**) Histogram of photocurrent variation of AuHgPt nanoalloys against 5 mM hydrogen peroxide at different voltages. (**b**) Light-enhanced current curves of the AuHgPt nanoalloys toward hydrogen peroxide.

In order to obtain higher light-enhanced response currents, the electrochemical response of AuHgPt nanoalloys to hydrogen peroxide was evaluated using chronoamperometry at different potentials in a 0.1 M phosphate buffer solution containing 1 mM hydrogen peroxide to select the optimal voltage for the experiments. From Figure 6a, it can be observed that after the addition of the light illumination, the change in current is not significant when the voltage is −0.1 V, whereas the current increases considerably when the voltage is −0.2 V and −0.3 V. Meanwhile, from the perspective of energy saving, the final selection of the experimental voltage was −0.2 V. The chronoamperometry method was used to further validate the light-enhanced electrochemical response of the AuHgPt nanoalloy-modified electrode to the electrochemical catalysis of hydrogen peroxide. This part focuses on the observation of the change of the current when hydrogen peroxide was added along with light irradiation. As can be seen in Figure 6b, the change in current is significantly increased by the addition of hydrogen peroxide, which indicates that the nanoalloys have a better electrocatalytic performance for hydrogen peroxide after the introduction of light.

**Figure 7:**
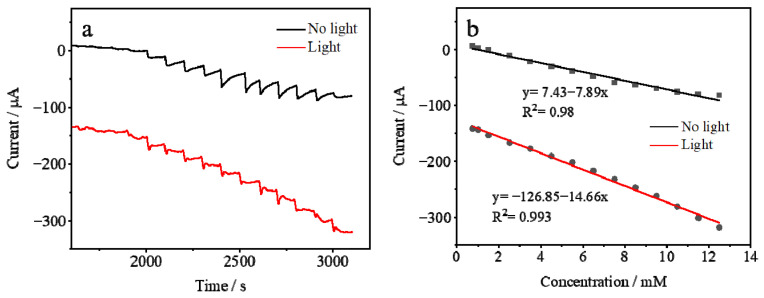
Amperometric response (**a**) and the calibration curves (**b**) of the AuHgPt/ITO electrode to the successive addition of hydrogen peroxide into 0.1 M phosphate buffer solution, applied potential −0.2 V, with (red line) and without (black line) light illumination.

Figure 7 shows the current response of the AuHgPt/ITO electrodes to different concentrations of hydrogen peroxide. Upon the addition of hydrogen peroxide to the system, the current increased rapidly and reached a steady state after about 4 s. The increase in current is attributed to the catalytic effect of the AuHgPt nanoalloy on hydrogen peroxide, as well as the effective diffusion of hydrogen peroxide in the solution, which enables it to fully participate in the electrode reaction, thus affecting the change in current. A comparison of the current changes before and after the introduction of light reveals that the current changes of the AuHgPt/ITO electrode with light are greater than those without light. This difference fully indicates that the light factor triggers the localized surface plasmon resonance of the nanoalloys, through which the sensitivity of the electrode to hydrogen peroxide is significantly improved. This enables it to respond more sensitively to the changes in the concentration of hydrogen peroxide, which is reflected in the magnitude of the current change. Further analysis illustrates that the increase in current showed a sufficiently linear relationship with the increase in hydrogen peroxide concentration. Under light conditions, the AuHgPt/ITO electrodes showed satisfactory detection performance with a detection range from 0.5 μM to 5 mM, a correlation coefficient R^2^ = 0.993 and a detection limit of 0.17 μM, as determined by the signal-to-noise ratio value of S/N = 3. It is noteworthy that the AuHgPt nanoalloy has a significantly lower detection limit for hydrogen peroxide compared to the hydrogen peroxide chemosensor in the absence of light irradiation and several other sensors using different precious metal nanomaterials (Table 1). This implies that the AuHgPt/ITO electrodes have a better detection ability under light irradiation in the same hydrogen peroxide detection situations, and can detect lower concentrations of hydrogen peroxide, which has a significant advantage and application potential in the relevant detection field.

**Figure 8:**
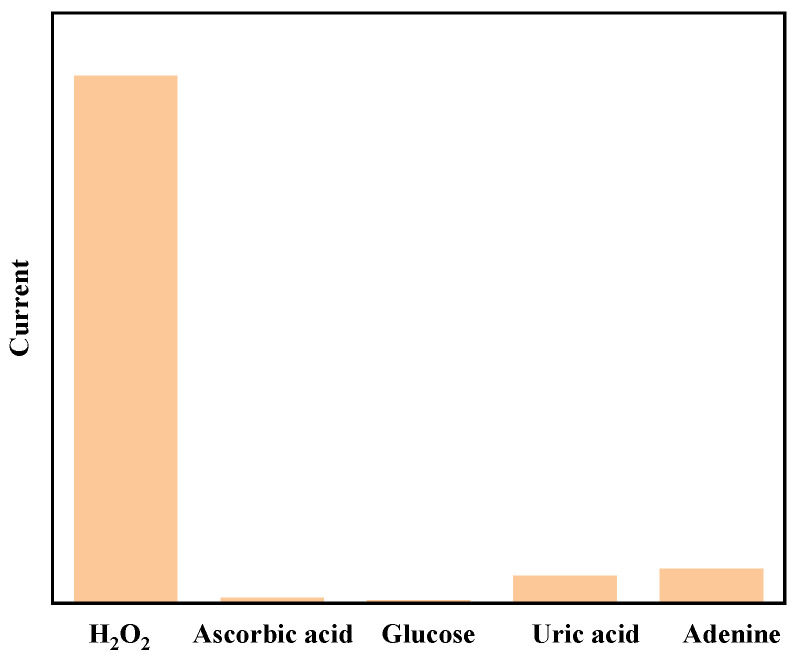
Amperometric response of the AuHgPt/ITO electrode towards the successive addition of hydrogen peroxide, ascorbic acid, glucose, uric acid and adenine in 0.1 M phosphate buffer solution with light illumination.

To fully evaluate the anti-interference capability of the AuHgPt/ITO electrodes, ascorbic acid, glucose, uric acid and adenine were examined under light conditions. The results shown in Figure 8 indicate that the electrode has a significant current response to hydrogen peroxide, but when these possible interfering substances were added to the system, the corresponding current change was very small. This result proves that the AuHgPt/ITO electrode is highly selective to hydrogen peroxide, and the interference of ascorbic acid, glucose, uric acid and adenine is almost negligible, which undoubtedly highlights the advantages of this electrode in the detection of hydrogen peroxide, enabling it to recognize and respond to hydrogen peroxide more accurately in complex real-world detection environments.

The reproducibility and stability of the AuHgPt/ITO electrodes were also evaluated. In terms of reproducibility, five electrodes prepared using the same method were simultaneously selected. Their electrochemical responses were detected and analyzed with a standard deviation of less than 5%, indicating that the performance of the electrodes prepared in different batches had a good consistency, which can ensure the reproducibility of the experimental results. After the electrode was placed in solution for one month, 1 mM hydrogen peroxide was added to the system under light conditions, and then electrochemical tests were carried out. It was found that the current response of the electrode was still able to reach about 85% of the initial state. Such test results demonstrate that the AuHgPt/ITO electrode is able to maintain a relatively stable performance, whether it is placed for a long time or repeatedly used, and its stability and reproducibility are at a good level. This lays a solid foundation for the subsequent application of the electrode in the field of practical testing and other related areas.

## 4. Conclusions

In summary, a facile and sensitive light-enhanced electrochemical sensor for the detection of hydrogen peroxide based on AuHgPt/ITO was successfully synthesized using electrochemical deposition and galvanic replacement methods. Structural and compositional characterization indicated that AuHgPt exists in alloy form. The electrochemical measurements showed that the AuHgPt/ITO electrode is extremely sensitive in detecting hydrogen peroxide under light conditions, which is mainly attributed to the addition of light to generate the LSPR effect in gold, thereby enhancing the response current for hydrogen peroxide detection. The linear range of the prepared sensor was from 0.5 μM to 5 mM with a detection limit of 0.17 μM. This work elucidates the potential of visible light-enhanced metal nanoalloy material-modified electrodes in the field of electrochemical sensors.

## Figures and Tables

**Table 1:** Comparison of the electrochemical performance of AuHgPt/ITO and other hydrogen peroxide sensors.

Material	Linear Range (μM)	Detection Limit (μM)	Ref.
Pt-TiO_2_ nanotube arrays	40–1.25 × 10^3^	4.0	[26]
Au–Ag NPs	0.5–2 × 10^3^	0.059	[27]
Au–Pd/graphene	5–11.5 × 10^3^	1.0	[28]
Au nanosheet	9.4–13 × 10^3^	1.62	[29]
AuHgPt	0.5–5 × 10^3^	0.17	This work

## Data Availability

Research data are available upon reasonable request by contacting the corresponding author.
